# Fall Prevention in the Elderly: A Community Intervention Project

**DOI:** 10.7759/cureus.116991

**Published:** 2026-09-27

**Authors:** Ana Rita Queirós, Ana M Gonçalves, Fátima L Gomes, Filipa S Couto, Inês G Castro, João Francisco Poças, Luís L Correia, Tiago Jorge Ribeiro

**Affiliations:** 1 Family Medicine, Unidade Local de Saúde de Santo António EPE, Porto, PRT; 2 Physical Medicine and Rehabilitation, Unidade Local de Saúde Gaia/Espinho, Porto, PRT

**Keywords:** accidental falls, community health services, elderly care & gerontology, fall prevention, geriatrics, preventive medicine, supervised exercise therapy

## Abstract

Introduction

Falls are the main cause of accidental morbidity and mortality among the elderly population, compromising autonomy and quality of life. This study, developed by a multidisciplinary team, aimed to implement and assess the feasibility and potential impact of a community-based intervention in Day Centers for the prevention of falls among older adults through a combination of health education, structured physical exercise, and recommendations addressing modifiable environmental and behavioral risk factors.

Materials and methods

A prospective single-group pre-post intervention study was conducted among older adults attending Day Centers. All older adults attending the participating Day Centers during the recruitment period were screened for eligibility. Participants who met the predefined inclusion criteria, without neurological or musculoskeletal conditions that would prevent participation, and who provided informed consent were included, resulting in a final sample of 31 participants. The intervention lasted 12 weeks and consisted of theoretical educational sessions with distribution of informational materials, as well as practical training focused on balance and muscle strengthening. The effectiveness of the intervention was assessed using pre- and post-intervention results of the Activities-specific Balance Confidence (ABC) Scale, Timed Up and Go (TUG) test, and 1-Minute Sit-to-Stand test.

Results

Results showed a slight improvement in participants' perceived balance, reflected by increased scores on the ABC scale (from 47.9% to 50.1%). Performance in the Sit-to-Stand test also improved (from 7.5 to 11 repetitions), suggesting gains in lower-limb strength. In contrast, performance on the TUG test declined, with the average completion time increasing from 17 to 19 seconds, indicating a less favorable change in mobility performance.

Conclusion

This study presents inherent limitations such as inter-individual variability among participants, adherence challenges, and contextual or environmental constraints for practical sessions. Despite these limitations, the project reinforces the importance of structured community interventions in empowering older adults to prevent falls, promoting active and safe aging. Future strategies should focus on optimizing adherence, tailoring interventions to the specific needs of geriatric populations, and extending the duration while simplifying the strategies used.

## Introduction

Falls are the most common domestic accident among older adults and the leading cause of accidental death after the age of 65 [1]. The natural aging process is associated with physiological changes such as visual and auditory deficits, decreased muscle strength, slower reaction time, and reduced balance, all of which increase susceptibility to falls [1].

The impact on this age group is significant: falls are one of the main causes of hospital admission and loss of functional independence [2]. In Portugal, between 2000 and 2013, six out of every 100 hospitalizations among older adults were attributed to falls, with an average hospital stay of 13 days and death occurring in approximately 6% of cases [2]. Beyond morbidity and mortality, the psychosocial impact is also noteworthy - particularly the fear of falling (ptophobia) - which leads to reduced mobility, social isolation, cognitive decline, depression, and an increased risk of institutionalization [3,4].

Current evidence shows that structured exercise programs are among the most effective interventions for preventing falls in the elderly. Multicomponent programs that integrate balance, gait, and strength training demonstrate consistent improvements in mobility and physical function, reducing fall rates by about 20-30% and the proportion of individuals experiencing at least one fall by approximately 15% [5,6]. The United States Preventive Services Task Force recommends performing these programs two to three times per week for at least 12 months, under supervision and with progression adjusted to individual capacity [1].

One widely studied example is the Otago Exercise Programme (OEP) - a structured program of strength and balance exercises developed for fall prevention in older adults, designed for home-based training [7]. Studies have shown that the OEP improves balance, gait, and functional performance, although the magnitude of benefit may vary in populations with greater frailty or comorbidities [8,9].

Several validated tools help assess fall risk and response to intervention. The Activities-specific Balance Confidence (ABC) Scale evaluates self-perceived balance in daily tasks of varying difficulty, making it useful for identifying fear of falling [10,11]. The Timed Up and Go (TUG) test allows for an objective assessment of mobility, gait ability, and balance, thereby inferring fall risk [12]. The 1-Minute Sit-to-Stand test quantifies lower-limb strength and endurance - parameters directly related to fall risk [13].

Despite the established effectiveness of exercise-based fall-prevention strategies, their implementation in community settings may be challenging, particularly among older adults with advanced age, multiple comorbidities, and variable functional capacity. There is a need for pragmatic interventions that translate evidence-based recommendations into accessible strategies that can be delivered through community institutions and integrated into older adults' daily routines. Day Centers may provide an appropriate setting for this purpose, allowing healthcare professionals to combine structured physical activity with health education and practical recommendations addressing modifiable environmental and behavioral risk factors.

In this context, we developed a comprehensive and multidisciplinary 12-week intervention for older adults attending two Day Centers, combining health education, structured physical exercise, and recommendations addressing modifiable environmental and behavioral risk factors. The primary objective was to assess the feasibility of implementing this community-based fall-prevention intervention and to describe changes in balance confidence and functional performance after 12 weeks, assessed using the ABC Scale, TUG test, and 1-Minute Sit-to-Stand test. Secondarily, the project aimed to promote active aging, autonomy, and safety in daily activities, while highlighting the potential role of collaboration between Primary Health Care and Physical Medicine and Rehabilitation in community-based fall prevention.

## Materials and methods

The project was conducted as a prospective, single-group pre-post intervention study in two Day Centers within the coverage area of the investigators’ Family Health Units. All older adults regularly attending the participating Day Centers during the recruitment period were screened for eligibility. Participants were eligible if they regularly attended one of the participating Day Centers, were able to safely participate in the proposed educational and physical activities, and provided written informed consent. Individuals with neurological or musculoskeletal conditions that could prevent safe participation in the intervention were excluded. Three individuals did not participate because they were unavailable, resulting in a final sample of 31 participants. The intervention was implemented over 12 weeks, between April and June 2025.

The intervention was organized chronologically into three main phases: baseline assessment, intervention, and post-intervention assessment.

Baseline assessment

Before the intervention, relevant sociodemographic and clinical data were collected from the Day Centers’ records and from the participants themselves. Participants underwent baseline assessment using the ABC Scale, TUG test, and 1-Minute Sit-to-Stand test. The ABC Scale is a 16-item self-report questionnaire assessing an individual's confidence in maintaining balance during a range of daily activities. The European Portuguese version was used and has been validated for the Portuguese population [14]. The TUG test assessed functional mobility, gait, and balance by recording the time required to stand from a chair, walk three meters, turn, return, and sit down. The 1-Minute Sit-to-Stand test assessed lower-limb muscular strength and endurance by recording the number of repetitions completed during one minute without using the armrests. Dyspnea and fatigue were assessed before and after the test using the Modified Borg Scale.

Intervention

The intervention combined health education, structured physical exercise, and education regarding modifiable environmental and behavioral risk factors. Two theoretical educational sessions were conducted at each participating Day Center, one at the beginning and one at the end of the intervention period (see Appendices section). The sessions were attended by older adults and Day Center staff and addressed the epidemiology, risk factors, consequences, and prevention of falls. Particular emphasis was placed on potentially modifiable intrinsic and environmental risk factors, including hazards in the home environment, inappropriate clothing and footwear, and factors related to daily activities. Informational leaflets containing checklists regarding home safety, clothing, and footwear were distributed, with recommendations intended to help participants identify and address potential fall hazards in their home environments.

A practical exercise component, adapted from the internationally validated OEP, was introduced and reviewed during each visit to each Day Center. Each visit lasted approximately one hour and included demonstration and supervised practice of the exercise program, clarification of participants' questions, and reinforcement of the educational content. The exercise program remained unchanged throughout the 12-week intervention and comprised three components: warm-up, lower-limb strengthening, and balance exercises. The warm-up consisted of two minutes of walking followed by controlled head and trunk rotations. Strengthening exercises included knee extension, knee flexion, hip abduction, heel raises, toe raises, and squatting movements, with 10 repetitions per exercise for two to three sets and two-minute rest intervals between sets. Balance exercises included sit-to-stand from a chair, single-leg stance for 10 seconds on each leg, and figure-eight walking. Participants were instructed to perform the exercise program twice weekly and were additionally encouraged to walk for 30 minutes twice weekly according to their functional capacity and under safe conditions.

The research team demonstrated and supervised the exercises, emphasizing safe execution and appropriate adaptation to participants' functional capacity. Participants with balance difficulties were advised to perform exercises in a controlled environment, either seated or with appropriate support, and to avoid any exercises that caused pain or discomfort. The intervention also aimed to empower Day Center staff to reinforce and repeat the exercise program with participants on a regular basis between visits. The same exercise program was maintained throughout the intervention period.

Post-intervention assessment

At the end of the 12-week intervention, the ABC Scale, TUG test, and 1-Minute Sit-to-Stand test were re-administered using the same procedures as at baseline. The assessments were performed by the same members of the research team under standardized conditions, using the same equipment and test procedures.

Data analysis

Changes between pre- and post-intervention assessments were summarized using descriptive statistics, including means, standard deviations, frequencies, and percentages. Data were entered and coded in Microsoft Excel (Microsoft Corporation, Redmond, Washington) prior to analysis. The research team set a target of at least a 20% improvement in the measures used to assess intervention effectiveness. Given the exploratory and feasibility-oriented nature of the project, the small sample size, and the absence of a control group, the analysis was limited to descriptive statistics. Changes between pre- and post-intervention assessments were summarized using means, standard deviations, frequencies, and percentages. No formal inferential statistical analysis was performed, as the study was not designed or powered to establish statistical efficacy or causal effects of the intervention.

Changes observed between pre- and post-intervention assessments on the ABC Scale, TUG, and 1-Minute Sit-to-Stand tests were summarized using descriptive statistics (means, standard deviations, frequencies, and percentages). The ABC Scale is a 16-item self-report questionnaire that assesses an individual's confidence in maintaining balance while performing a range of daily activities, with the aim of identifying perceived balance limitations and risk of falls. The European Portuguese version was used by the authors and is a validated tool for the Portuguese population [14].

In the TUG test, the participant should begin the test seated in a chair. Upon instruction to start, the participant should stand up from the chair and walk at a comfortable and safe pace to a marker positioned three meters from the starting point. After crossing the line, the participant should turn around, walk back to the chair, and sit down again. The time required to complete the entire task should be recorded.

According to Podsiadlo and Richardson [12], TUG completion times of less than 20 seconds indicate normal mobility, times over 20 and of less than 30 seconds indicate good mobility and the ability to go outdoors independently without the need for walking assistance, whereas times over 30 seconds indicate impaired mobility and the need for assistance with ambulation.

The 1-Minute Sit-to-Stand test is a performance-based measure designed to assess lower-limb muscular strength and endurance in adults. The participant is instructed to repeatedly stand up from and sit down on a chair without using the armrests for support for one minute, at a pace that feels comfortable and safe, until instructed to stop. The total number of repetitions completed should be recorded, as well as the levels of dyspnea and fatigue before and after the test, using the Modified Borg Scale.

These tools were chosen for their validity, simplicity, and relevance to older populations: the ABC provides a qualitative measure of fall risk, while the TUG and 1-Minute Sit-to-Stand test offer quantitative measures of mobility, balance, and lower-limb strength - capacities directly linked to autonomy and frailty risk.

The research team set a target of at least a 20% improvement in the scales used to assess intervention effectiveness.

All participants provided written informed consent, and all data were handled confidentially using a coded, password-protected database. The study was approved by the coordinators of the participating Family Health Units and Day Centers. Ethical approval was granted by the Santo António/ICBAS Ethics Committee (ref. 2024.230), and authorization was obtained from the Board of Directors of ULS Santo António.

Table 1 presents the project’s implementation, monitoring, and evaluation schedule.

**Table 1:** Chronological timeline of study activities USF – Family Health Units; ULS – Local Health Unit

	February 2025	March 2025	April 2025	May 2025	June 2025	August 2025
Favorable opinion of the study by the Ethics Committee of ULS Santo António	X					
Coordination with the Day Centers		X				
Baseline assessment and initial intervention			X			
Mid-intervention follow-up				X		
Final intervention session and post-intervention assessment					X	
Data analysis						X

## Results

A total of 34 individuals attending the participating Day Centers were considered during the recruitment period, of whom three did not participate because they were unavailable. The final sample therefore comprised 31 participants, 24 (77.4%) of whom were female. All included participants completed the post-intervention assessment.

The sample included users aged 56 to 95 years, with a mean age (± SD) of 79.4 ± 9.6 years. The most common comorbidities were hypertension (n=22, 71.0%), dyslipidemia (n=17, 54.8%), degenerative osteoarticular disease (n=10, 32.3%), type 2 diabetes mellitus (n=8, 25.8%), ophthalmological disease (n=7, 22.6%), and dementia (n=6, 19.4%). The distribution of all comorbidities in the sample is presented, divided according to the chapters of the ICPC-2 (International Classification of Primary Care, 2nd edition), in Figure 1.

**Figure 1:**
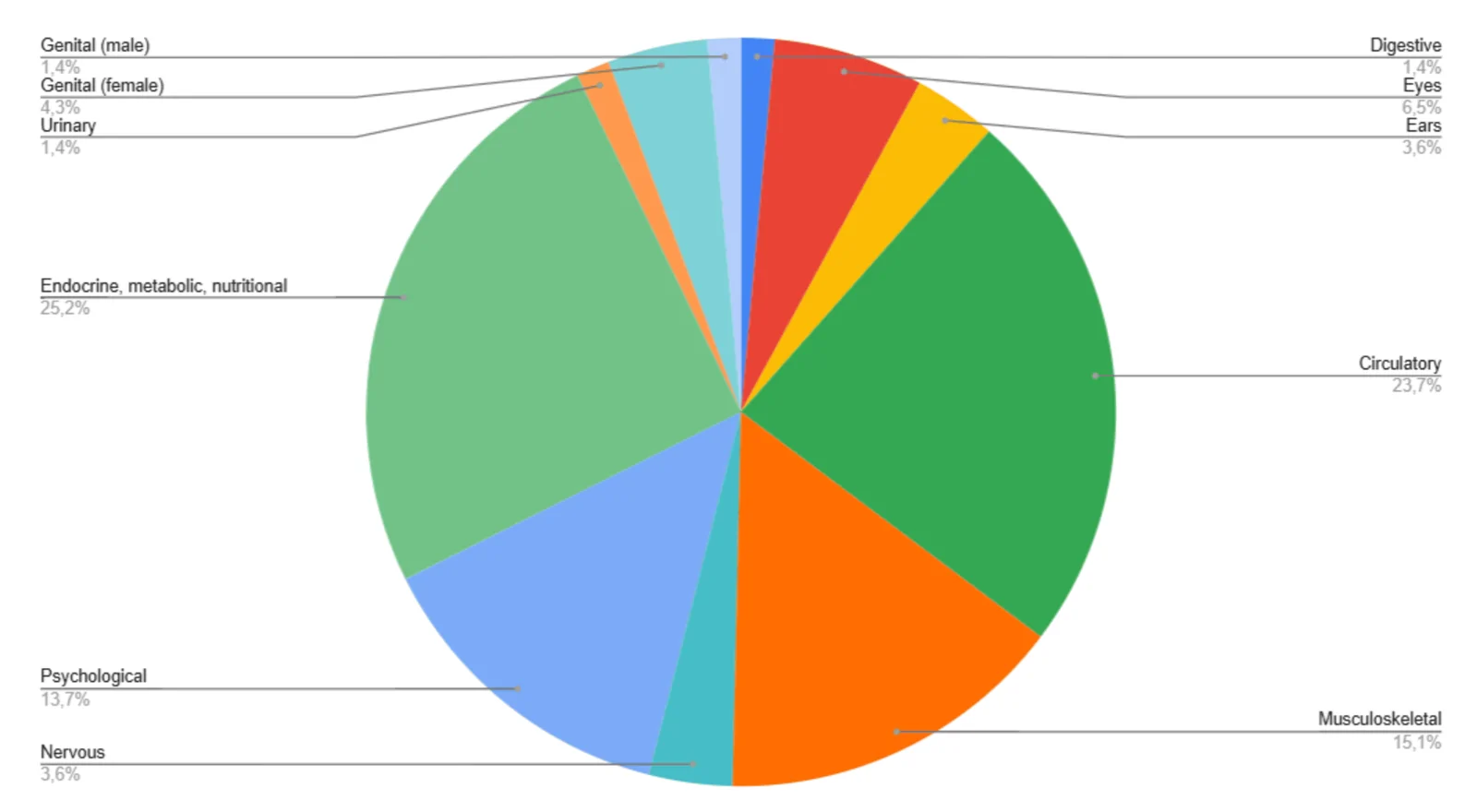
Distribution of comorbidities in the elderly sample according to ICPC-2 chapters (International Classification of Primary Care, 2nd edition)

Regarding performance on the applied tests, it was found that the average score on the ABC Scale increased from 47.9% to 50.1% at the end of the intervention. The average time to complete the TUG test increased from 17 to 19 seconds, and the average number of repetitions in the 1-Minute Sit-to-Stand test increased from 7.5 to 11 after the intervention. The evolution of the results of the three pre- and post-intervention assessments is presented in Table 2.

**Table 2:** Evolution of the results studied in the pre- and post-intervention periods ABC Scale – Activities-specific Balance Confidence; TUG – Timed Up and Go

Evaluation	Pre-intervention (April 2025)	Post-intervention (June 2025)	Absolute variation	Relative variation
ABC Scale (points)	47.88	50.13	+2.25	+4.7%
TUG Test (seconds)	17.00	19.00	+2,00	+11.8% (worst performance)
1-Minute Sit-To-Stand Test (repetitions)	7.50	11.00	+3.50	+46.7%

## Discussion

This project, developed by a multidisciplinary team of Family Medicine and Physical Medicine and Rehabilitation physicians, enabled the description of changes observed following a structured 12-week community-based fall-prevention intervention among older adults attending two Day Centers.

The results were heterogeneous, reflecting the diversity of the participants and the inherent challenges of functional assessment in older populations.

Descriptive analysis showed an increase in balance confidence, reflected by a 4.7% increase in the ABC score, although this did not reach the predefined target. This finding may indicate that changes in the perception of postural safety and fear of falling occur gradually and may require longer intervention periods to become clinically meaningful in institutionalized older adults. Similar findings have been reported in previous intervention studies, where improvements in balance confidence were generally smaller than improvements in objective physical performance [15]. Fear of falling is influenced not only by physical capacity but also by psychological, behavioral, and environmental factors, which may require prolonged exposure to exercise and repeated successful mobility experiences before measurable improvements are observed [16].

In parallel, the mean time required to complete the TUG test increased by 11.8%, indicating an observed decline in functional mobility during the study period. This observation may reflect fluctuations in clinical status, such as pain, fatigue, intercurrent illness, or the natural progression of frailty, as well as differences in adherence and participation in the intervention sessions. Previous studies have shown that mobility measures such as the TUG are particularly sensitive to acute health events, pain, fatigue, and day-to-day functional variability in frail older adults [17,18]. Consequently, short-term deterioration in TUG performance does not necessarily contradict concurrent improvements in muscle strength following exercise interventions.

In contrast, performance in the 1-Minute Sit-to-Stand test improved by 46.7%, exceeding the predefined target and indicating enhanced lower-limb functional capacity. This pattern is consistent with evidence from systematic reviews demonstrating that multicomponent exercise programs combining resistance and balance training improve lower-limb strength and functional performance in community-dwelling older adults, even when changes in other mobility outcomes are more modest [5,17]. Taken together, these findings suggest that improvements in muscle performance may be observed earlier, whereas changes in mobility and balance confidence may require interventions of greater intensity and duration.

The observed improvements in balance confidence and Sit-to-Stand performance suggest that community-based exercise interventions may contribute to functional benefits in older adults, including populations characterized by advanced age and multiple comorbidities. However, the absence of improvement in TUG performance indicates that mobility and dynamic balance may be more complex domains, potentially requiring longer and more intensive interventions.

The available literature is heterogeneous in both methodology and outcomes. Systematic reviews indicate that fall-prevention programs incorporating muscle strengthening and balance exercises may reduce both the incidence and severity of falls [19]. Our findings are broadly consistent with studies reporting improvements in muscle strength following short-term programs combining aerobic activity, strengthening exercises, and balance training. Similarly, other programs have reported improvements in muscle strength, balance, and fall prevention following weekly physical activity workshops conducted over 12 weeks [20].

Several methodological factors may help explain the variability observed across outcomes. First, the intervention duration was limited to 12 weeks, which may not have been sufficient to produce measurable changes in domains that are generally more resistant to modification, such as dynamic balance and mobility. Previous studies suggest that programs lasting at least six months and incorporating progressive balance challenges produce more consistent improvements in mobility and fall outcomes than shorter interventions [1,5]. Second, the study included a relatively small sample of participants with advanced age and multiple chronic conditions, factors that may influence responsiveness to exercise interventions and contribute to substantial inter-individual variability.

The relatively small final sample of 31 participants also limited the statistical power of the study and the generalizability of the findings to broader populations of older adults. Together with the absence of a control group, these limitations preclude causal inference regarding the observed pre- to post-intervention changes and reinforce the exploratory and feasibility-oriented nature of the project.

Important limitations related to assessment procedures should also be considered. The functional measures used (ABC Scale, TUG, and 1-Minute Sit-to-Stand) require comprehension of instructions, sustained attention, physical effort, and participant cooperation. Although these instruments have been validated for use in older populations, their administration can be demanding. Auditory, visual, or cognitive impairments, as well as fluctuations associated with chronic diseases, may have influenced participant performance. Furthermore, all assessments were conducted sequentially during the same session, which may have contributed to fatigue and affected performance, particularly in mobility-related tests. These factors should be considered when interpreting the findings and highlight the importance of selecting assessment tools that are appropriate for the cognitive and physical characteristics of the target population.

Despite these limitations, this project demonstrated the feasibility of implementing a low-cost, multidisciplinary, community-based intervention in a Day Center setting. These findings are aligned with current recommendations advocating community-based, multidisciplinary fall-prevention programs delivered through primary care and community institutions as feasible strategies to promote healthy aging [6]. The observed changes in balance confidence and lower-limb functional performance support further investigation of similar programs. From a clinical perspective, these findings highlight the potential role of Primary Health Care and Physical Medicine and Rehabilitation services in the development of fall-prevention strategies tailored to the needs and capabilities of older adults.

Future studies should include larger samples, longer intervention periods, and assessment strategies designed to minimize fatigue and communication barriers. Improving participant adherence through more interactive sessions, individualized follow-up, and engagement strategies may also enhance program effectiveness. In addition, implementing similar interventions earlier in the aging process, when individuals have greater functional reserve, may contribute to primary fall prevention and help reduce the occurrence of first-time falls.

## Conclusions

This community-based fall-prevention intervention was feasible to implement among older adults attending Day Centers. After 12 weeks, participants showed a modest improvement in balance confidence and a notable increase in lower-limb functional strength, as measured by the ABC Scale and 1-Minute Sit-to-Stand test, respectively. In contrast, TUG performance did not improve. Given the small sample size and absence of a control group, these findings should be interpreted descriptively and do not establish intervention effectiveness. Larger controlled studies with longer follow-up are needed to further evaluate the impact of community-based multidisciplinary fall-prevention interventions.

## Figures and Tables

**Figure 2:**
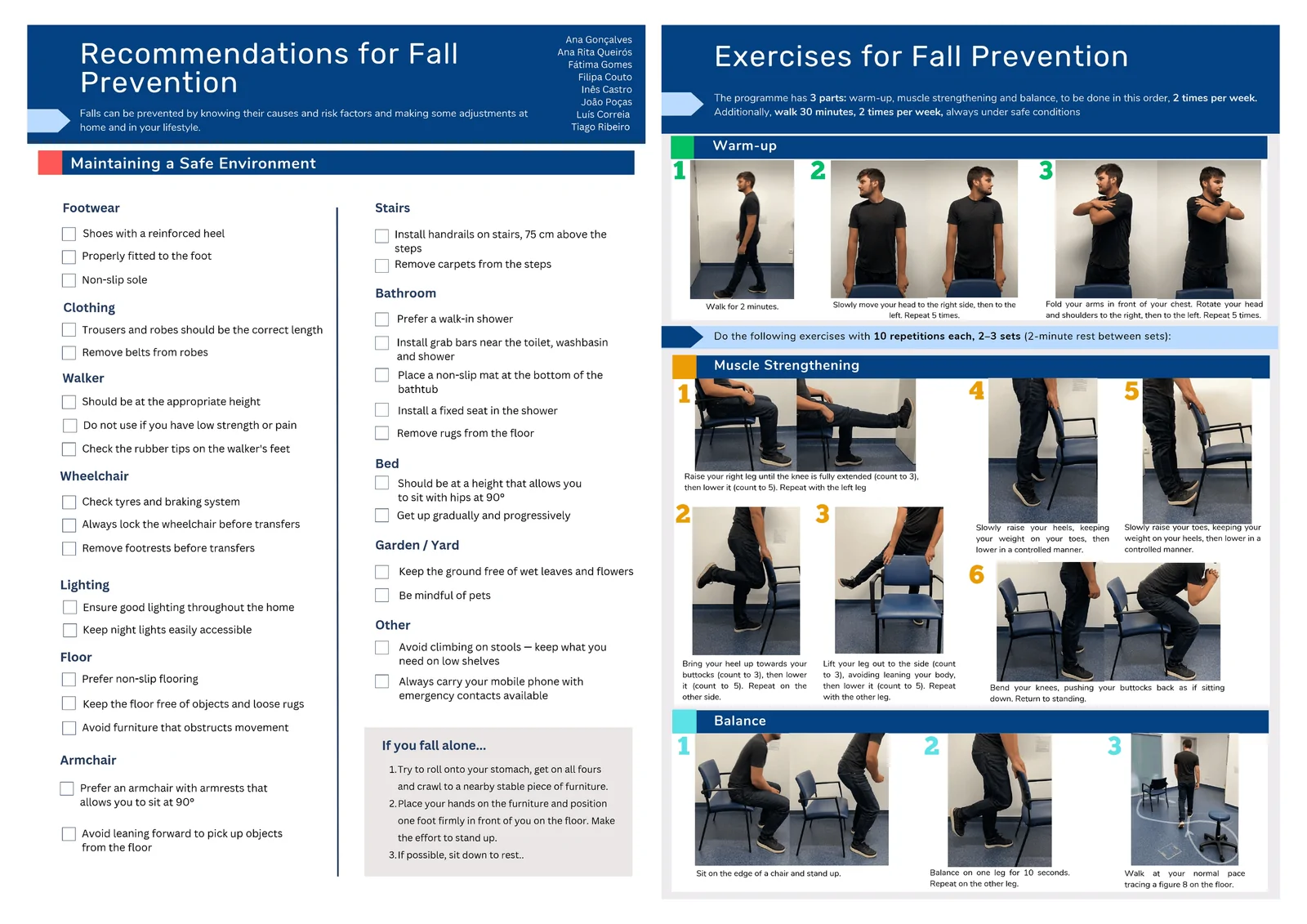
Educational leaflet providing general fall-prevention recommendations and a home-based exercise program to be performed regularly (in Portuguese)

## Appendices

Educational materials to be distributed in Day Centers

As previously mentioned, the authors provided educational and practical training sessions, providing participants with the material shown in Figure 2.

## References

[1] Nicholson WK, Silverstein M, Wong JB (2024). Interventions to prevent falls in community-dwelling older adults: US Preventive Services Task Force recommendation statement. JAMA.

[2] (2025). Norma no 008/2019: Prevenção e Intervenção na Queda do Adulto em Cuidados Hospitalares [Internet]. https://normas.dgs.min-saude.pt/2019/12/09/prevencao-e-intervencao-na-queda-do-adulto-em-cuidados-hospitalares/.

[3] Gai J, Gomes L, Jansen De Cárdenas C (2009). Ptophobia: the fear of falling in elderly people (Article in Portuguese). Acta Med Port.

[4] Watson BJ, Salmoni AW, Zecevic AA (2016). The use of the Morse Fall Scale in an acute care hospital. Clin Nurs Stud.

[5] Sherrington C, Fairhall NJ, Wallbank GK (2019). Exercise for preventing falls in older people living in the community. Cochrane Database Syst Rev.

[6] Colón-Emeric CS, McDermott CL, Lee DS, Berry SD (2024). Risk assessment and prevention of falls in older community-dwelling adults: a review. JAMA.

[7] Thomas S, Mackintosh S, Halbert J (2010). Does the 'Otago exercise programme' reduce mortality and falls in older adults? A systematic review and meta-analysis. Age Ageing.

[8] Cheng M, Ni J, Liu F, Wang S (2026). Optimal type and dose of exercise to improve fall behavior in older adults: a systematic evaluation and network meta-analysis. Ageing Res Rev.

[9] Dong M, Liu X, Choi Y, Li N (2025). Effects of Otago exercise program and aquatic exercise on fall risk in older adults: a systematic review. Arch Gerontol Geriatr.

[10] Powell LE, Myers AM (1995). The Activities-specific Balance Confidence (ABC) Scale. J Gerontol A Biol Sci Med Sci.

[11] Myers AM, Fletcher PC, Myers AH, Sherk W (1998). Discriminative and evaluative properties of the activities-specific balance confidence (ABC) scale. J Gerontol A Biol Sci Med Sci.

[12] Podsiadlo D, Richardson S (1991). The timed "Up & Go": a test of basic functional mobility for frail elderly persons. J Am Geriatr Soc.

[13] Bohannon RW, Crouch R (2019). 1-minute sit-to-stand test: systematic review of procedures, performance, and clinimetric properties. J Cardiopulm Rehabil Prev.

[14] Branco PS (2010). Validation of the Portuguese version of the “Activities-Specific Balance Confidence Scale”. Rev Soc Port Med Física E Reabil.

[15] Tinetti ME, Richman D, Powell L (1990). Falls efficacy as a measure of fear of falling. J Gerontol.

[16] Scheffer AC, Schuurmans MJ, van Dijk N, van der Hooft T, de Rooij SE (2008). Fear of falling: measurement strategy, prevalence, risk factors and consequences among older persons. Age Ageing.

[17] Beauchet O, Fantino B, Allali G, Muir SW, Montero-Odasso M, Annweiler C (2011). Timed Up and Go test and risk of falls in older adults: a systematic review. J Nutr Health Aging.

[18] Barry E, Galvin R, Keogh C, Horgan F, Fahey T (2014). Is the Timed Up and Go test a useful predictor of risk of falls in community dwelling older adults: a systematic review and meta-analysis. BMC Geriatr.

[19] El-Khoury F, Cassou B, Charles MA, Dargent-Molina P (2013). The effect of fall prevention exercise programmes on fall induced injuries in community dwelling older adults: systematic review and meta-analysis of randomised controlled trials. BMJ.

[20] Sanchez M, Vidal JS, Bichon A (2023). Impact of a public open-access community-based physical activity and fall prevention program on physical performance in older adults. Eur J Public Health.

